# Primary Mediastinal Neuroendocrine Tumor: A Case of Atypical Carcinoid

**DOI:** 10.7759/cureus.12853

**Published:** 2021-01-22

**Authors:** Konstantinos Kosmas, Ioannis Vamvakaris, Eirini Klapsinou, Eleni Psychogiou, Dimitra Riga

**Affiliations:** 1 Cytology Department, General Hospital of Thoracic Diseases of Athens “Sotiria”, Athens, GRC; 2 Pathology Department, General Hospital of Thoracic Diseases of Athens “Sotiria”, Athens, GRC; 3 Department of Cytology, Diagnostic and Therapeutic Center of Athens “Hygeia”, Athens, GRC

**Keywords:** pmnet, case report, primary mediastinal neuroendocrine tumor, immunohistochemistry

## Abstract

Primary mediastinal neuroendocrine tumor (PMNET) is an extremely rare clinical entity and few cases have been described in the literature. Here, we report a histologically confirmed rare PMNET case of a 66-year-old male patient with a mass detected in the anterior upper mediastinum by chest high-resolution computed tomography (HRCT). Early detection and surgical intervention of this neoplasm are critical for long term survival, though the tumor is associated with a dismal outcome.

## Introduction

Lung cancer was the most frequently diagnosed cancer (2.093.876 total cases) and the leading cause of cancer death (1.761.007 total cancer deaths) worldwide in 2018 for both sexes combined [1]. Neuroendocrine tumors (NETs) are epithelial neoplasms with predominant neuroendocrine differentiation that arise in most organs of the body [2] including the lung, thymus, gastrointestinal tract (GI), and ovary. The lung is, following the GI tract, the second most common site for NETs [3] accounting for 25% of all primary lung neoplasms [4] and 1%-2% of all invasive lung malignancies [5]. The most common NETs in the mediastinum include thymic neuroendocrine carcinomas, aorticopulmonary and paravertebral paragangliomas, and ectopic or supernumerary parathyroid tumors. Primary NETs of the mediastinum have been a source of controversy in the literature due to the ongoing debate about their origin, nomenclature, and classification. Their prognosis is usually poor despite a number of treatment options [6]. Primary mediastinal neuroendocrine tumor (PMNET) is an exceptionally rare occurrence with limited cases that can be accounted for in literature. Thus we report for review, the case of a 66-year-old man with a primary atypical carcinoid (AC) tumor of the anterior upper mediastinum diagnosed histologically.

A part of this case report was presented as a brief abstract in the 36th Balkan Medical Week on September 25-26, 2020 in Bucharest, Romania [Abstract: Riga D, Vamvakaris I, Kosmas K, Megas P, Papadopoulou A, Psychogiou E. IM10. Primary mediastinal neuroendocrine tumor (PMNET). A case of atypical carcinoid (AC)].

## Case presentation

**Figure 1:**
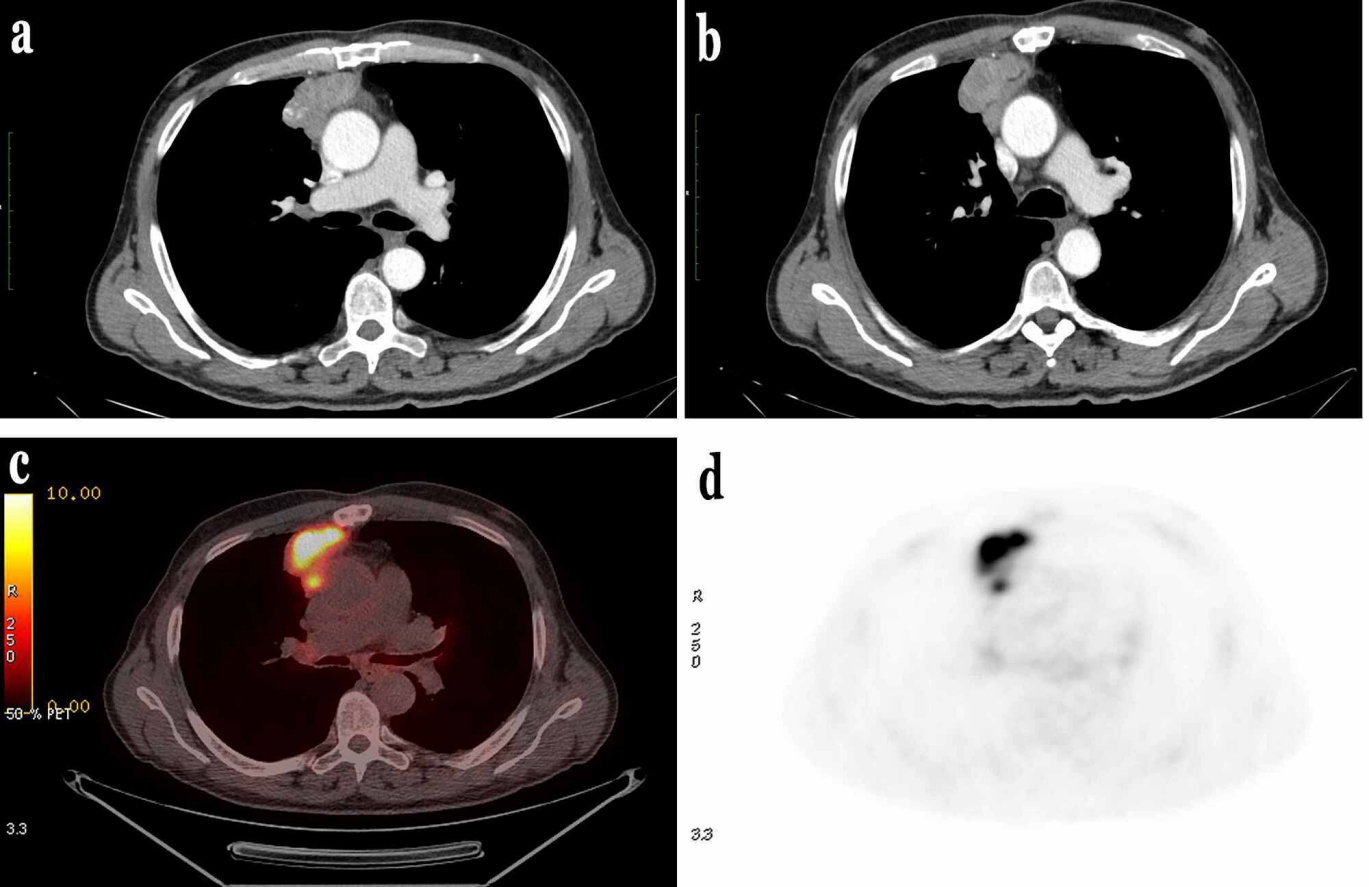
Imaging findings. (a,b) Chest CT images demonstrate a tumor mass in the anterior upper mediastinum.  (c,d) FDG-PET scan images demonstrate high uptake in the mediastinal mass without metastasis.

A 66-year-old male smoker, presented in the outpatient department complaining about dyspnea and fatigue for over two weeks. High-resolution computed tomography (HRCT) scan revealed a tumor mass measuring 8x5x3.3 cm localized in the anterior upper mediastinum. Fluorodeoxyglucose-positron emission tomography (FDG-PET) scan demonstrated high uptake in the mediastinal mass without metastases (Figure 1).

**Figure 2:**
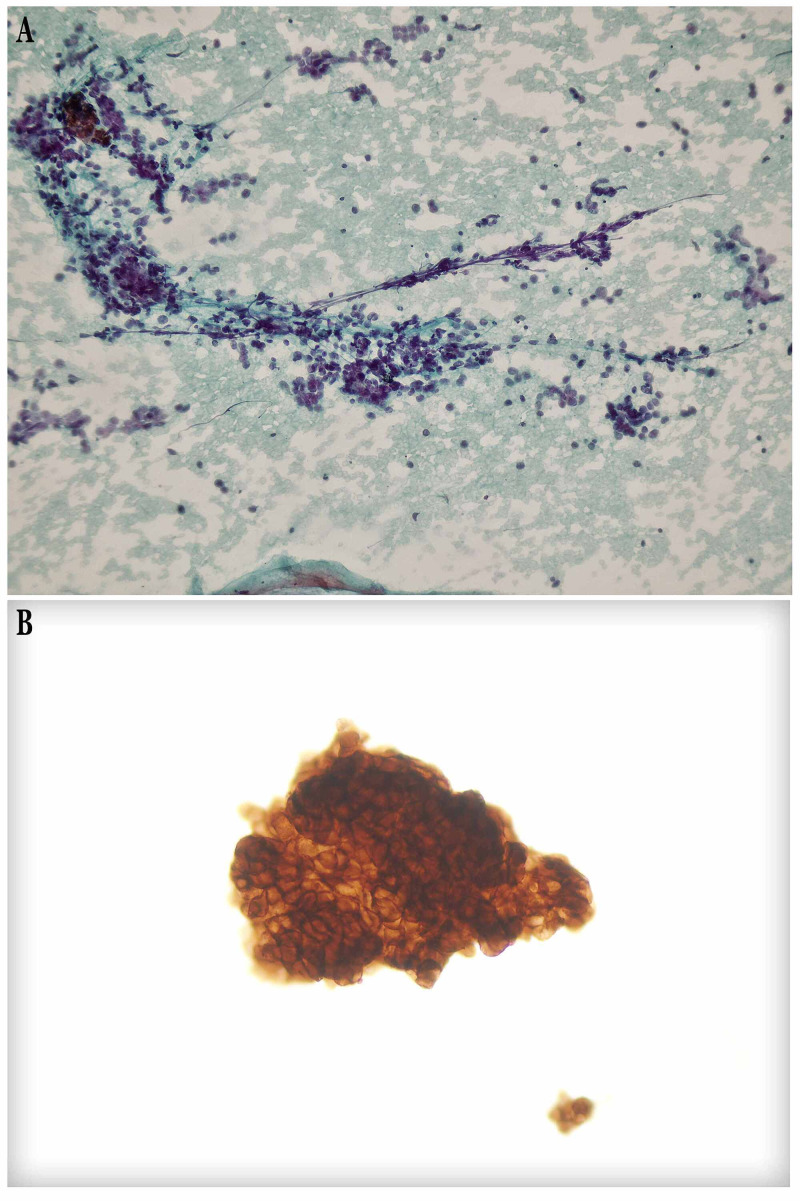
Cytopathological features. (A) Numerous uniformly small-medium round to oval in shape tumor cells, with scant and pale eosinophilic cytoplasm and relatively small bland nuclei with finely granular chromatin pattern (Papanicolaou stain ×200). (B) CD56 immunocytochemical positivity (×200).

The patient underwent endobronchial ultrasound-guided transbronchial needle aspiration (EBUS-TBNA). The cytological examination revealed numerous uniformly small-medium round to oval in shape tumor cells, with scant and pale eosinophilic cytoplasm and relatively small bland nuclei with finely granular chromatin pattern. Neither necrosis nor mitotic figures were found. Immunocytochemical staining was positive for neural cell adhesion molecule (NCAM/CD56) and negative for thyroid transcription factor-1 (TTF-1) while Ki-67 labeling index was not estimable (Figure 2).

**Figure 3:**
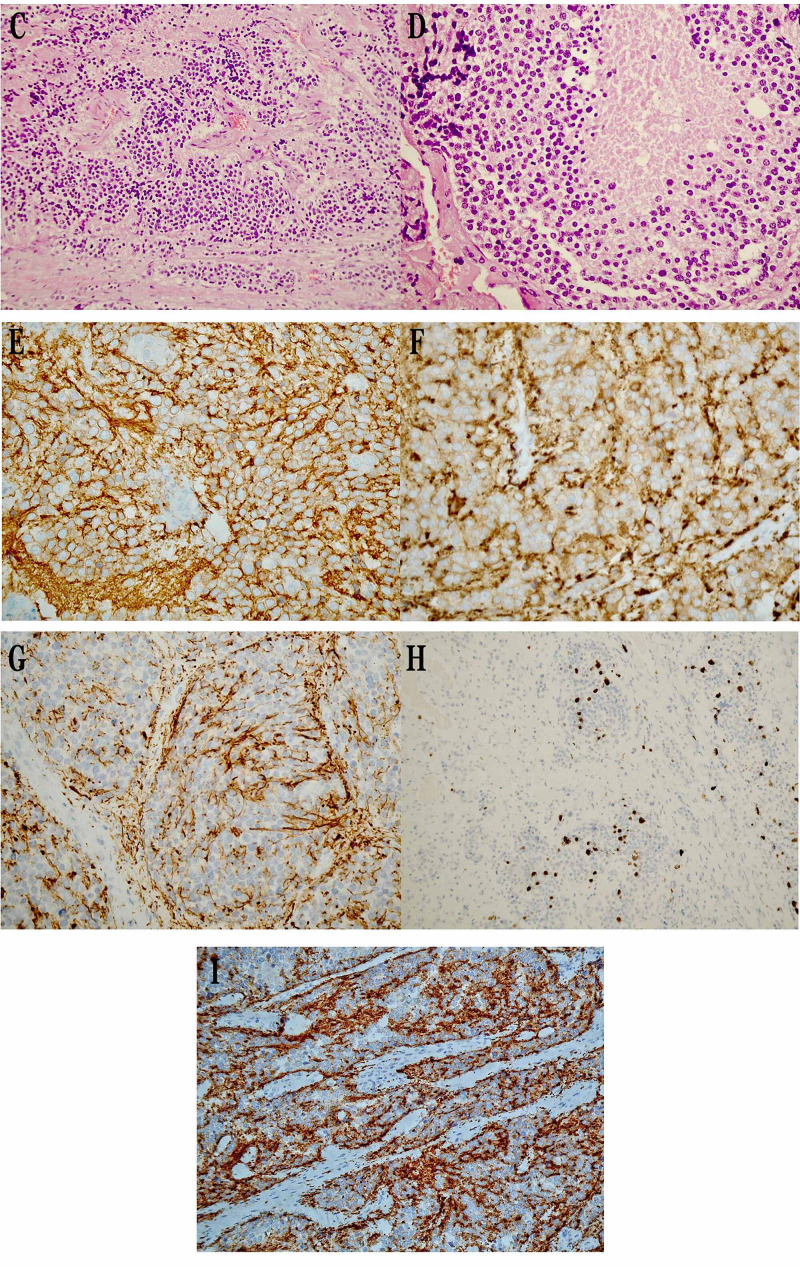
Histopathological features. (C) Neoplastic cells arranged in trabeculae, nests, and rosette-like formations. Necroses were observed (Hematoxylin and Eosin stain x200). (D) Medium-sized neoplastic cells with oval normochromatic or hyperchromatic nucleus and coarse chromatin (Hematoxylin and Eosin stain x400). (E) CD56 positivity (immunohistochemistry ×400). (F) Synaptophysin positivity (immunohistochemistry ×400). (G) CK18 positivity (immunohistochemistry ×200). (H) Overexpression of Ki-67 labeling index (immunohistochemistry x200). (I) Chromogranin A positivity (immunohistochemistry ×400).

The cytological diagnosis was neoplastic tumor with neuroendocrine features. Surgical excision was proposed and the tumor was radically excised. Histopathologically, the neoplastic cells were medium-sized, with oval normochromatic or hyperchromatic nucleus and coarse chromatin, arranged in trabeculae, nests, and rosette-like formations. Necroses were observed and the number of mitoses was eight per 2 mm2 [10 high-power fields (HPF)]. Neither thymic tissue nor bronchogenic epithelium were found after thorough sampling throughout the lesion. Immunohistochemically the tumor cells were leukocyte common antigen (LCA) (-), p40(-), TTF-1(-), CD30(-), CD5(-), CD1a(-), CD117(-), placental alkaline phosphatase (PLAP) (-), alpha-fetoprotein (AFP) (-), Cytokeratin19 (CK19) (-), Cytokeratin18 (CK18) (+), CD56(+), CD99(-), synaptophysin(+), chromogranin A(+) and Ki-67(+) in 20% of the cells (Figure 3).

Resection margins were tumor free. Based on the above data, PNET, lymphoma, adenocarcinoma, small cell carcinoma (SCC), rhabdomyosarcoma, and germ cell tumor were excluded. We concluded the diagnosis of PMNET-AC (intermediate grade), according to World Health Organization (WHO) 2015. Six months after surgery and without receiving any adjuvant treatment, the patient remains free of disease under clinical follow-up.

## Discussion

The current 2015 WHO classification of tumors of the lung, pleura, thymus, and heart provides diagnostic criteria for NETs based on the histopathologic characteristics such as cell morphologic features, architectural growth patterns, mitotic rate, and the presence or absence of necrosis. Tumors with these neuroendocrine morphologies are divided into high-grade malignancies, SCC and large cell neuroendocrine carcinoma (LCNEC), low-grade typical carcinoid (TC), and intermediate-grade AC, primarily according to the number of mitoses, the presence or absence of necrosis, and the detection of immunohistochemical markers such as chromo­granin A, synaptophysin, and CD56 [7,8]. Clinically, patients may be usually asymptomatic. When present, symptoms may be due to the compression or invasion of adjacent intrathoracic structures or associated with the tumor's ability to produce hormones or cytokines [6]. There are striking differences in the clinical settings of low and intermediate-grade neuroendocrine tumors versus high-grade neuroendocrine carcinomas (NECs). The former occurs in younger patients (mean age, 50 years), with no inclination for sex or smoking history, whereas the latter occurs predominantly in older male patients (mean age, 65 years) and almost invariably heavy smokers [4]. Carcinoid tumor's initial symptoms are cough, hemoptysis, chest pain, wheezing, dyspnea, and recurrent pneumonia [5,9]. In contrast, our patient was a smoker, over 65 years old with no initial symptoms. AC has significantly more aggressive biological behavior than TC with the frequency of distant and nodal metastases ~20% and ~50% respectively [4] and five-year survival of 76% [10]. Although surgery is the treatment of choice for ACs [5], the relapse rate for carcinoid tumors ranges between 5% and 30% [9] and is higher (65%) in ACs and patients with mediastinal node involvement [9,10]. Therefore, adjuvant treatment with chemotherapy and/or thoracic radiation therapy is recommended, even though their efficacy has not been adequately demonstrated [9]. Several hormonal receptors, e.g., somatostatin, have been described in NETs as having diagnostic, prognostic, and therapeutic implications due to their high receptor-binding affinity [11]. Somatostatin analogs, such as octreotide and similar derivatives, e.g., lanreotide, have been used for their antisecretory effects but they have shown moderate efficacy in controlling symptoms [5,9]. An accurate diagnosis in cytology samples requires conscientious attention to morphological and immunohistochemical features. TC and AC cannot be reliably distinguished in cytology, however, AC may be suspected if necrosis or mitoses are present. If a high proliferation activity is seen by Ki-67 labeling index, this may be helpful to exclude low-grade NETs. In addition, Ki-67 antigen can effectively be used in cell block preparations to avoid misdiagnosis of poorly differentiated NET, whereas this procedure is not reliable for distinguishing TC from AC on conventional smears [5]. In cytologic specimens of our case, all the cytomorphological and immunocytochemical features lead us to the diagnosis of a neoplastic tumor with neuroendocrine features. In the present case, neither evidence of thymic nor parathyroid involvement nor sustentacular cells were identified within the lesion, thus, this tumor was diagnosed as true PMNET-AC.

## Conclusions

The accurate diagnosis of PMNET is critical for the patient’s treatment and the possibility of long-term survival, though the tumor is usually associated with a dismal outcome. Furthermore, the diagnosis of atypical carcinoid by cytology alone is difficult, regardless of the cytopathologist’s expertise, and therefore requires attentive histological evaluation and immunohistochemical assays.
